# Special Issue: Nano-Catalysts and Nano-Technologies for Green Organic Synthesis

**DOI:** 10.3390/molecules16021452

**Published:** 2011-02-09

**Authors:** Angelo Nacci, Nicola Cioffi

**Affiliations:** 1 Department of Chemistry, Università degli Studi Aldo Moro, Via Orabona 4, 70126-Bari, Italy; 2 CNR – ICCOM, Department of Chemistry, Università degli Studi Aldo Moro, Via Orabona 4, 70126-Bari, Italy

**Keywords:** nanoparticles, catalysis, green chemistry

## Abstract

Catalysis by transition-metal nanoparticles has undergone an explosive growth during the past decade. This special issue presents the general trends in the current research in this field, the present situation concerning scope and limitations, as well as the future perspectives. Original contributions are also presented on the applications of nano-catalysts to the green synthesis.

Transition-metal nanoparticles (NPs) are attracting a great deal of attention in almost any scientific and technological field, including catalysis, where nanoscale materials are becoming more prevalent in a wide range of applications such as fuel conversion, pollution abatement and fine chemical production (*viz*. pharmaceuticals, agrochemicals, flavours, fragrances, electronic chemicals, *etc*).

The synthesis and structural characterization of transition metal nanoparticles are also topics of interest in which chemists strive to control chemical composition, particle size (quantum-size effects can play an important role), morphology, internal structure (such as alloyed or layered mixed-metal systems), as well as crystallographic structure, in order to achieve a better understanding of structure-activity trends. It can be expected, in fact, that the effective control of the catalyst key features, either when used in the homogeneous phase, or when anchored on heterogeneous supports, will also allow for successful applications at the industrial scale.

An increasing interest is also devoted nowadays to properly exploit the high activity and selectivity of nanocatalysts in order to develop greener and waste-minimized processes. From the Green Chemistry standpoint, new nanocatalysts must be designed to operate under environmentally friendly (for instance phosphine-free) conditions or in neoteric green solvents (e.g. ionic liquids, supercritical fluids, fluorous phases, water and so on). This allows their easy removal from the reaction media, and guarantees a more prolonged recycling with high efficiency. 

This special issue of *Molecules* is focused on the use of nanocatalysts and/or nanotechnologies in several catalytic organic reactions based on Green Chemistry principles (atom-economy, dematerialization, energy saving, raw material diversification, green solvents, *etc*.). Four excellent reviews are presented here, illustrating the general trends in current research. The state of art is given on various types of nano-sized metal catalysts such dendrimer-encapsuled Pd nanoparticles, prepared by “click synthesis” and very active at very low (almost “*homeopathic”*) loadings, palladium-containing copolymer micelles, soluble palladium-containing polymers, and Pd nanoparticles dispersed in several types of ionic liquids. Representative examples are also presented on the dependence of the catalytic activity on nanoparticle shape, or on the use of bi-, tri-, and multi-metallic nanoparticles. A wide set of traditional carbon-carbon and carbon-heteroatom bond forming reactions such as Heck, Suzuki, Sonogashira, Stille, Ullmann, heterocyclizations, carbonylations, cyanations, Michael additions etc, are proposed as benchmark for the nano-sized metal catalysts here discussed.

In addition, six selected original contributions are also presented, reporting multidisciplinary studies on nanoscale materials (viz. nanoparticles, nanocomposites, polymer-encapsuled NPS, nanorods, nanotubes, nanotemplates *etc*.) and their chemical functionalization, including their development/synthesis, together with the analytical chemical and morphological characterization, as well as organic synthesis applications.

Innovative topics of these articles are the synthesis of recyclable NP-supported rhodium catalysts for hydrogenations, the preparation of PdO nanoparticle supported on carbon nanotubes and graphene oxide useful for nitrophenols reduction, the use of Pd colloids on zirconia as recyclable catalysts for the Heck, Suzuki and Ullmann homo-couplings, the catalytic performance of ceria nanorods in the oxidations of hydrocarbons, the hydrogen generation promoted by a CuO/ZnO-ZrO_2_ nanocatalyst in the reforming of methanol for fuel cell applications. Finally, the use of mesoporous aluminosilicates in the acid-catalysed conversion of saccharides into furanic aldehydes, provides an excellent example of the conversion of renewable biomass sources into fine chemicals.

We wish to thank all the authors for their outstanding cooperation and support to this issue, and we hope the reader will find useful information about this cutting-edge research field. 

